# Supplementary material for: Are carbon emissions trading and green financial instruments synergistic? -Comprehensive quantitative research based on content analysis
Source: PLoS One. 2024 Mar 7;19(3):e0298601. doi: 10.1371/journal.pone.0298601 (PMC10919601; doi:10.1371/journal.pone.0298601)
Supplement: S1 Data — (ZIP) [file pone.0298601.s001.zip › Data/地级市Z_F工作报告.zip!/╝¬┴╓/┴╔╘┤╩╨/└┤╫╘╣1⁄2╓┌║┼í╛┬φ┐╦╩2╛▌═°í┐_2012.docx]

各位代表：

　　现在，我代表市政府，向大会作政府工作报告，请予审议，并请市政协各位委员提出意见。

　　一、2011年工作回顾

　　2011年，市政府认真贯彻落实省委、省政府和市委的决策部署，深入践行科学发展观，统筹实施 “三动”、“三化”战略，加快推进“三个转型”，克服宏观经济形势严峻、区域竞争压力加大等不利因素，圆满完成了市六届人大四次会议确定的目标任务，实现了“十二五”良好开局。预计地区生产总值完成500亿元，增长22%；全口径财政收入完成33亿元，增长17.6%；全社会固定资产投资完成400亿元，增长35.1%；社会消费品零售总额完成127亿元，增长18%；外贸进出口总额完成9500万美元，增长54%；城镇居民人均可支配收入和农民人均纯收入达到18600元和7200元，分别增长11.6%和13.9%；人口自然增长率控制在2‰；城镇登记失业率控制在3.6%；单位GDP能耗降低率和主要污染物减排量完成省下达指标。

　　经济转型取得新进展。坚持投资拉动、项目带动、创新驱动，促进总量扩张、结构优化和产业升级。实施3000万元以上项目252个，其中新开工项目120个，竣工项目71个。鑫达钢铁技术改造、利源铝业交通型材、方大锻造锻压生产线、富奥泵业三泵扩建等项目扎实推进。巨峰生化乙酸乙酯、中聚新能源锂电池、麒鸣牧业羊血液综合开发、汇丰新能源汽车电机及控制器等项目正式投产。三海新天地购物广场、亨通物流园区等现代服务业项目基本完工。高精铝加工、纺织袜业、钢铁铸造等产业基地初具规模，汽车零部件、建筑机械和新能源等产业集群正在形成。推动辽源经济开发区二次创业，启动寿山、东西孟工业园区建设。三次产业比重达到10：58：32。预计规模以上工业总产值、增加值、利润完成850亿元、230亿元、21亿元，分别增长43.4%、27.1%、39.1%。产值超亿元企业达到192户。《辽源市资源枯竭型城市转型规划》顺利通过国家评审。

　　城市转型进入新阶段。城市规划体系基本建立，城市建设管理水平不断提高，打造吉林省中南部区域中心城市迈出坚实步伐。辽白经济一体化、南部新城开发正式启动，东丰西城区、白云新城建设快速推进。吉草高速辽源段建成通车，辽西高速征地拆迁基本结束。一环路全线贯通，改造、新建矿北大街等11条街路和仙城西路桥、辽河广场、仙城景观带。东辽河城区段2.8公里综合治理工程竣工，库河联调工程、污水处理厂升级改造快速推进。矿山环境综合治理和东辽河流域综合治理项目全面启动，辽源国家矿山公园子项目博物馆和主题广场开工建设。辽河源水患问题逐步得到解决。城市生活垃圾处理场主体完工。“数字辽源”基础网络建设加快推进。“五城联创”取得阶段性成果，省级园林城创建两年任务一年完成，市容环境和交通秩序进一步改善。

　　“三农”工作实现新发展。强化科技支撑，提高农机化水平，加强农田水利基本建设，粮食总产量达到35亿斤。改扩新建标准化牧业小区89个，肉蛋奶产量增长12 %以上。棚膜作物面积发展到3万亩，蔬菜种植面积达到15万亩。市级以上农业产业化龙头企业达到67户，金翼、麒鸣、鹏翔、睿康等重点企业发展势头强劲。完成36个新农村省级示范村项目建设，启动19个样板村（区）建设，评选出12个辽源“最美乡村”。农村环境卫生整治和生活垃圾集中处理试点取得积极成果。各项惠农政策全面落实，发放“四项补贴”3.28亿元。农村各类合作经济组织发展到969个，增长109%；农民劳务收入实现6.8亿元，增长17%。县域经济发展取得可喜成绩。

　　改革开放迈出新步伐。医药卫生体制改革、城乡户籍制度改革取得阶段性成果。事业单位人事制度改革、行政审批权相对集中改革、财政管理体制改革不断深化。街道社区管理体制改革、土地收储制度改革、集体林权制度配套改革全面启动。民营企业股份制改造扎实推进，重点企业上市步伐加快。组织赴欧洲、北美、深港、长三角等地招商活动12次，接待各类考察洽谈团组105批次，参加第七届东博会取得丰硕成果。先后与宁波均胜、大连美罗、深圳粤海、芬兰拉普兰、美国莱克森等大企业成功合作，签约项目52个，落地大项目12个。引进域外资金279亿元，增长18.8%；实际利用外资1.59亿美元，增长25.5%。探索建立“互保池”融资合作模式。增发10亿元企业债通过国家初审。第二届PE高峰论坛、第三届房交会等活动富有成效。

　　人民群众实惠不断增多。年初承诺的80件惠民实事全面完成，当年投入达到37亿元。保障性安居工程征收13600户，开工111万平方米，回迁10352户，分配廉租房909套。改造农村泥草房1788户。80万平方米既有建筑节能改造及热源、热网建设全面完成。改造老旧小区41个，亮化“黑楼道”820栋。市区24小时供水率提高7.3个百分点。天然气置换达到1.2万户。创建国家级创业型城市取得突破性进展，新增民营企业1024户，新增个体工商户1.1万个，带动就业3.1万人。开发公益性岗位905个，“零就业”家庭动态为零。城乡养老保险实现全覆盖，医疗和工伤保险实现“一卡通”。企业退休人员基本养老金和失业保险金分别增长16%和40%，城乡医保报销比例提高到70%，患者住院费用和平均药价降低30%。建立物价上涨和社会保障标准联动机制，城乡低保标准分别增长10%和19%。设立企业家扶贫助残基金、教育资助基金，849名贫困大学生得到资助。收回市殡仪服务中心经营权，降低了居民殡葬费用。市结核病院异地新建并投入使用。

　　社会各项事业不断进步。科技创新、科技成果转化取得积极成效，60个项目列入国家和省级科技计划，争取无偿资金额、项目申报成功率居全省前列。申请发明专利18项，新增国家级高新技术企业3户，新增省级科技企业11户，已有65户企业与51所高校和科研机构建立合作关系。高新技术产业增加值占规模以上工业增加值比重达到22%。教育布局日趋合理，在全省率先取消义务教育阶段择校。教育质量不断提高，高考、中考连创佳绩。中小学校舍安全工程基本完成，办学条件明显改善。北方矿业职教集团正式成立，职业教育园区建设稳步推进。“活力辽源”行动计划深入实施。显顺琵琶走进维也纳金色大厅，全民健身中心向社会开放，东丰农民画馆落成，乡镇综合文化站实现全覆盖。加快建设二战盟军高级战俘营展览馆、辽源游泳馆、博纳数字电影城、红旗文化广场、贺龙体育公园等工程。成功举办首届“东辽河文化艺术节”，送戏下乡、广场演出等群众性文体活动丰富多彩。编制完成旅游总体规划，增加3条地接旅游线路，聚龙潭、寒葱顶等景区建设扎实推进。我市被国家人口计生委、国家人社部评为人口计生工作先进市，并被确定为全国人口计生综合改革示范市。市档案馆建成投入使用，军分区异地新建及公安、司法、民政业务用房建设有序推进。民族宗教、环境保护、外事侨务、残疾人就业康复等工作得到加强。

　　社会管理创新不断深入。积极探索社区网格化管理模式，建立健全社会矛盾调处体系，调解各类纠纷4万余件，调解成功率达到98%。扎实开展“积案化解年”和“局长大接访”活动，信访积案息访率达到77%，局长接访化解率达到89%。安全生产工作得到加强，连续三年获得省政府安全生产目标责任制考核一等奖，受到国家安监总局表彰。平安辽源建设成效显著，建立智能交通卡口系统，新增平安监控探头4835个，刑事案件发案率下降5%，群众安全感满意度位居全省前列，荣获全国见义勇为城市奖。食品药品监管工作扎实有效，得到国务院督查组和省政府充分肯定。“六五”普法全面启动。连续27年无重大森林火灾。

　　政府自身建设不断加强。自觉接受人大及其常委会的法律监督、工作监督和政协的民主监督，高度重视社情民意，42件代表建议和155件政协提案全部办结。坚持科学民主决策，建立分工负责的重点工作推进机制，领导干部“一岗五责”全面落实，“百日攻坚”等工作扎实有效。推进行政审批大提速，开通重大项目审批直通车，实行企业“宁静日”制度，经济发展软环境进一步改善。切实落实党风廉政建设责任制，确保行政权力阳光运行，政府形象进一步提升。

　　一年来，市政府注重把握工作大局，抓实发展第一要务，坚定民生第一目标，履行稳定第一责任，辽源转型发展呈现新局面；注重突破重点难点，抓住主要矛盾，积极应对挑战，全力攻坚克难，经济发展、城市面貌、干部群众精神状态发生新变化；注重统筹协调发展，坚持经济与社会统筹，发展与民生统筹，城市与县域统筹，改革与稳定统筹，政府各项工作取得新成果；注重强化开放创新，不断解放思想，创新实践，上争下促，内引外联，辽源跨越发展注入新动力；注重效能政府建设，倡导尽责、务实、高效、廉政的服务理念，建立重点工作督导落实的推进机制，政府执行力和公信力得到新提升。

　　成绩来之不易，发展鼓舞人心。这是市委正确领导，市人大、市政协和社会各界监督支持的结果，凝聚着全市人民的智慧和力量。在此，我代表市政府，向全市各族人民，向各民主党派、工商联、人民团体、无党派人士、离退休老同志，向驻辽部队、武警官兵、中省直单位，向关心和支持辽源发展的各界朋友，致以崇高的敬意和衷心的感谢！

　　同时，我们也清醒地看到存在的不足。主要表现在：辽源经济基础仍很薄弱，结构性矛盾比较突出，县域突破任务十分艰巨；发展面临的资源环境等刚性约束增强，土地、资金矛盾仍很突出，部分重大项目受政策调整影响未能如期建设；文化软实力和影响力不强，城市文化特色不够鲜明；居民收入增长尚未与经济发展同步，城乡贫困人口比例仍然较高，一些民生问题解决得还不够好；少数干部的服务意识、办事效率、执行能力与形势变化、职责使命和人民群众期待不适应，作风建设、软环境建设有待进一步加强。对此，我们将采取得力措施，认真加以解决。

　　二、2012年工作安排

　　2012年，是我市实现跨越发展十分关键的一年。面对世界经济下行风险加大的趋势，稳增长、控物价、调结构将是明年国家宏观调控的主要任务。发达地区产业渐次向欠发达地区转移，长吉图开发开放和沈阳经济区层次提升，为辽源快发展、大发展既提供了难得机遇，也提出了严峻挑战。对此，我们必须审时度势，积极应对，抢抓机遇，合力攻坚，推动辽源各项事业加快发展。

　　政府工作要以党的十七届六中全会、中央经济工作会议、省委九届十二次全会、市六次党代会精神为指导，深入贯彻落实科学发展观，坚持“三化”统筹和“三动”并举，以加快转型、全面转型为工作主线，突出总量扩张、产业升级、改善民生、县域突破、招商引资、繁荣文化、提升城市功能、加快城镇化进程等重点任务，协调推进经济、政治、文化、社会、生态建设，积极争创国家转型示范城市，奋力开创辽源跨越发展新局面。

　　经济社会发展主要预期目标是：地区生产总值增长25%，达到625亿元；规模以上工业增加值增长30%，达到300亿元；全社会固定资产投资增长30%，达到520亿元；全口径财政收入增长25%以上；社会消费品零售总额增长22%；外贸进出口总额增长20%；城镇居民可支配收入和农民人均纯收入增长15%；城镇登记失业率控制在3.9%以内；人口自然增长率控制在2.3‰以内；城镇化率达到58.4%；单位GDP能耗降低率和主要污染物减排量完成省下达指标。重点抓好以下工作：

　　（一）加快结构调整，努力构建现代产业体系

　　把经济结构战略性调整作为全面转型的主攻方向，注重自主创新，增加有效投资，加快产业升级，促进可持续发展。一要强力推动重大项目建设。计划实施3000万元以上项目329个，其中新开工152个，竣工130个，积极谋划和储备一批支撑作用强、税收贡献大、综合效益好的重点项目。加快培育新材料、新能源、电子信息等战略性新兴产业，重点实施利源铝业交通型材、世捷铝业板带箔材、麦达斯铝业新能源汽车车体、中聚新能源扩能改造、博大伟业生物制药、美罗生物健康保健品等项目。改造提升装备制造、冶金建材、纺织袜业等传统产业，重点实施鑫达钢铁技术改造、均胜汽车零部件、国泰矿山机械等项目。二要着力打造特色园区和产业集群。在辽源经济开发区打造高精铝加工、新能源、汽车零部件、纺织袜业、生物制药和电子产业园；在东西孟工业区打造碳纤维制品、绿色食品、高档家俬产业园；在寿山工业区打造高新技术、现代物流产业园；在渭津工业区打造建筑机械、建筑材料产业园；在东丰经济开发区打造梅花鹿产业园；在白云工业区打造冶金铸造产业园；在东辽工业集中区打造矿山装备、大型锻造产业园。坚持集约节约用地，提高投资强度，强化节能减排，严把项目准入标准。加快退城进区步伐，实施富迪纺织、辽河纺织等重点企业搬迁。东丰经济开发区晋升省级开发区，推动辽源经济开发区升级国家级开发区。产值10亿元以上企业达到13户，其中矿业集团超过50亿元，鑫达钢铁公司力争达到100亿元。三要大力推进服务业跃升。积极扶持现代物流、信息服务、文化旅游、房地产开发等服务业加快发展，致力打造区域物流中心和特色旅游城市。重点实施玉圭园水上游乐场、粤海电子商贸园区、中鑫粮食物流园区、苏宁购物中心、融展汽贸城等项目，积极发展民俗游、红色游、工业游、生态游，加快形成市中心区、辽河半岛、开发区商圈，规划建设南部新城、北部城区商圈。结合新区开发与旧城改造，开工建设辽河北岸城市综合体、辽河半岛东侧高档商住等项目，促进房地产市场健康发展。四要切实强化科技和人才支撑。加强产学研合作，鼓励自主创新，争创省级创新型城市。启动15个重大科技攻关项目，培育10户省级以上高新技术企业、创新型企业和科技型企业，建立5个省级以上研发中心和工程中心，建设10个大专院校和科研院所的科技成果转化基地，打造一批拥有自主知识产权和核心技术的名牌产品。建立院士工作站，创建省级科技孵化器，大力培养和引进高层次、高技能人才。高新技术产业增加值占比达到25%，科技贡献率达到50%以上。五要全面建设企业服务平台。组建中小企业服务联盟，建成中小企业服务大厦。完善中小企业投融资体系，深入开展“银企保”对接活动，加快行业联盟和企业“互保池”建设。扶持微型企业初始创业，建成5个以上标准化创业孵化器。全面提升中小企业成长性，实施企业家队伍培育工程和企业素质提升工程。鼓励、支持和引导非公有制经济加快发展，积极营造企业健康发展的良好氛围，致力打造全民创业最活跃和行政服务效率最高地区。

　　（二）加快新区开发，积极推进新型城市建设

　　充分发挥规划的引领作用，坚持高质量建设、高标准管理，加快构建以城带乡、城乡互动、全域融合的一体化发展格局。一要加快中心城区扩容。加快推进南部新城建设，博物馆、图书馆、群众艺术馆、规划科技展览馆主体实现封闭，启动行政中心和职教园区建设，形成两纵两横路网框架，实施河道整治和绿化美化配套工程。加快完善北部新城、寿山新区基础设施，打造城区东北部路网，完成东裕路建设。加速推进辽白经济一体化，实现公交、电信同城化。二要加快基础设施建设。全力推进辽西、集双高速和辽长、四松铁路辽源段“两高两铁”项目。实施库河联调工程，启动北城区供水改造和城市二次供水改造，做好“引松入辽”工程前期工作。完善“三环四纵六横”路网体系，新建改造路桥10条，建设面积25万平方米。继续推进智能电网和“气化辽源”工程。启动粪便无害化处理项目，力争建成垃圾发电项目。三要深入推进“五城联创”。省级卫生城具备申报条件，国家级环保模范城和省级生态城建设加快实施，国家级文明城创建工作取得重要进展。完成龙首山、向阳山、北山亮化工程，推进渭津河、梨树河河口景观化，打造亮化美化精品街区。强化城市综合执法，建立常态运行机制。加快推进“数字辽源”建设，全面提升城市智能化管理水平。四要大力加强生态建设。实施碧水蓝天工程，加大治理力度。全面推进矿山环境综合治理和东辽河流域及上游水源地综合整治，建成国家矿山公园博物馆、主题广场，建设辽源国家矿山湿地公园和杨木水库、东辽河湿地公园。着力解决半截河水患和污染问题。实施第二个“十年绿化美化辽源大地规划”，打造东辽河二环内“绿色走廊”。逐步搬迁市区洗煤场、储煤场，严控企业污染排放，进一步提高城区空气质量。

　　（三）加快县域突破，全面增强县域经济实力

　　坚持用工业化思维谋划全市农业大发展，切实加强“三农”工作。做好发展精细种植业、精品畜牧业、精深加工业的大文章，启动建设粮食高产核心区、畜牧产业精品区、园艺特产示范区、农畜产品精深加工区、生态农业观光区等现代农业园区。大力实施农田水利基本建设，改善农业生产条件，确保粮食稳产高产。新建标准化牧业小区30个，新建标准化蔬菜大棚1000栋，提高本地农副产品自给水平。做大做强农业产业化龙头企业，支持金翼集团、麒鸣牧业、鹏翔牧业、东丰药业、睿康生物等重点企业打造产业链和开发新产品。培育壮大合作经济组织，扶持发展牛、羊、猪、鸡、梅花鹿、柞蚕、食用菌等特色镇、专业村和规模养殖户，多渠道增加农民收入。扎实推进新农村样板村（区）建设，深入实施农村环境综合整治。

　　坚持工业化和城镇化“双轮驱动”，加快园区开发和新城建设，吸纳要素集聚，培育立县产业，实现县域经济升级晋位。加快东丰西城区和东辽北城区建设，规划打造容纳10万人口的白云新城，推进横道河、那丹伯、三合、渭津等重点镇建设，谋划启动建安、金州组团。支持企业和项目向小城镇规划区集聚，引导农民向小城镇和中心村集中，配套生产性服务业和生活性服务业，积极培育立县产业和骨干企业。两县新增产值5亿元以上企业不少于10户，当年落地亿元以上项目不低于10个，主要经济指标增幅超过30%。

　　（四）加快实施富民工程，切实保障和改善民生

　　坚持以人为本、民生优先，决不与民争利，认真办好惠民实事，让转型发展成果更多地惠及全市人民。扎实推进全民创业，争创国家级创业型城市，新增创业5000人，新增就业3.2万人，开发公益性岗位500个以上。全面开展新型农村社会养老保险和城镇居民养老保险试点，强力推进非公企业参保扩面，积极实施残疾人助保政策，适时启动厂办大集体改革，继续提高企业退休人员养老金待遇。做好国有关闭破产企业退休人员参加医疗保险工作，启动城镇居民基本医疗保险门诊统筹和生育保险市级统筹。严格执行最低工资制度，不断增加居民收入，继续提高城乡低保标准。出台扶贫、扶弱、助孤、助残政策措施，完善残疾人、老年人、留守儿童保障和服务体系，扶持各类扶弱济困基金健康发展，启动县区整村扶贫开发计划。强力推进保障性安居工程建设，棚户区改造征收房屋1.4万户，开工建设150万平方米，煤矿棚户区改造回迁4800户。继续实施“暖房子”工程，加快热源、热网及配套设施建设，完成既有建筑节能改造90万平方米。深入开展住宅小区治理工程，改造36个老旧小区，建设3个精品小区。市区24小时供水率提高15个百分点。天然气置换1.2万户。积极发展便民交通，延伸公交线路，增加运营班次，完成仙城客运站和公交枢纽中心建设。完善市场供应保障体系，稳定消费价格水平，大力抓好“菜篮子”工程。

　　（五）加快汇聚发展活力，进一步扩大改革开放

　　全面推进重点领域和关键环节改革。实施事业单位分类改革，深化医药卫生体制改革，完成文化、广电、新闻出版机构改革，推进街道社区管理体制改革。加快投融资体制改革，扩大股权融资、企业债券、中期票据等直接融资，探索土地质押和经营模式，组建城投公司，运作新区开发建设。做好增发10亿元企业债工作。既要适度举债，为加快发展提供必要的资金支撑，又要有效控制和防范政府债务风险。坚决杜绝非法集资。争取民生银行设立驻辽分支机构。农村信用社申报组建农商行。继续组织PE走进辽源活动，促进企业与基金对接。德春米业实现上市，金翼蛋品、麒鸣牧业等企业上市取得突破性进展。

　　实施招商引资攻坚战，注重引资与引智结合，促进外部资源内化，形成全方位、多层次、宽领域的对外开放格局。突出主导产业和重点领域，锁定珠三角、长三角、环渤海等重点区域，实施定向招商、驻点招商、专业招商、以商招商。推动驻美和驻欧工作站建设，邀请知名商会、财团和央企来辽考察洽谈，积极筹办首届国际棉袜展销会。强化招商合力，落实招商责任，探索招商路径，健全考评体系，加强跟踪服务，进一步优化投资环境。推动本地企业总量扩张、效益提升和管理升级，促进域内外企业交流合作，积极靠大联强，加快低成本扩张。更加注重招商实效，全面提高项目履约率和资金到位率。争取引进域外资金348亿元，增长25%；实际利用外资1.9亿美元，增长20%，签约落地重大项目不少于50个。加快“省级铝型材产品出口基地”、“省级鞋袜产品出口基地”建设，外贸进出口总额突破1亿美元。

　　（六）加快文化发展繁荣，不断完善公共服务

　　坚持面向基层、服务百姓，积极发展公益性文化事业和经营性文化产业，加快打造特色文化名城。逐步完善文化基础设施，博纳数字电影城、红旗文化广场投入使用，两县图书馆、文化馆及乡镇综合文化站、街道社区文化中心建设加快推进。深入实施“活力辽源”行动计划，举办首届国际琵琶艺术节，积极组织送戏下乡和广场演出活动，广泛开展全民健身运动，全面启动十七届省运会场馆建设。大力挖掘梅花鹿、农民画、满族剪纸等地域文化资源，积极打造辽河源、皇家鹿苑、龙首山古城、反法西斯遗址群落等特色文化品牌，加快发展乒乓球培训、琵琶培训和电视剧制作等文化产业，培育壮大创意、动漫、传媒、数字制作等新型业态，稳步推动印刷包装产业园区建设，不断提高文化产业增加值占GDP的比重。

　　切实加大教育投入，提高义务教育均衡化水平，加快普及高中段教育，实施学前教育三年行动计划。扎实推进职教园区建设，打造东北地区较有影响力的技能型人才培养输出基地。构建综合医院、专科医院、基层医疗服务格局，完善市县乡村医疗卫生服务网络，不断提升医疗卫生服务水平。大力促进民族团结和宗教和谐。启动中国／联合国人口基金第七周期生殖健康／人口与发展项目。

　　（七）加快推进社会管理创新，精心维护稳定和谐

　　不断完善社会矛盾纠纷调解工作格局，充分发挥人民群众和社会组织依法参与社会管理的积极性，严格落实信访、安全生产责任制和责任追究制，致力打造平安辽源、法治辽源、活力辽源、和谐辽源。坚持群防、技防、自防多措并举，加强“天网”工程建设，严厉打击犯罪活动，进一步增强群众安全感。高度重视安全生产工作，切实加大重点行业整治力度，坚决遏制较大以上事故发生。加大“局长大接访”工作力度，变群众上访为干部下访，积极回应群众合理诉求，着力解决信访突出问题，有效化解各种矛盾和风险隐患。推进食品药品安全体系建设，健全食品药品监管机制，保障人民群众饮食用药安全。完善防灾减灾应急预案，加快应急指挥平台建设，积极预防和妥善处置突发公共事件。加强双拥共建和国防后备力量建设，不断巩固和发展军政军民团结。

　　三、进一步加强政府自身建设

　　要顺应新形势、新任务和人民群众的新期待，进一步提高政府执行力、公信力。

　　要努力建设规范有序的法治政府。自觉接受人大及其常委会的法律监督、工作监督和政协的民主监督，认真倾听百姓呼声，高度重视社情民意，加大代表建议、委员提案办理力度。坚持科学民主决策，完善政府议事规则，重大事项实行公众参与、专家论证、风险评估、合法性程序审查和集体讨论决定。建立规范高效的工作流程，推进财政预算、公共资源配置、重大建设项目等领域的政府信息公开。

　　要努力建设为民执政的责任政府。始终把群众利益作为一切工作的出发点和落脚点，爱民动真情，惠民见实效。畅通群众诉求、利益协调、权益保障渠道，及时回应群众关切，有效化解民生难题，纠正损害群众利益的不正之风。坚持问政于民、问需于民、问计于民，从群众中汲取智慧、获取力量，依靠群众把好事办好、难事办成。

　　要努力建设高效勤政的效能政府。进一步解放思想、创新思维、更新理念，不断提高领导科学发展、改善民生和破解复杂问题的本领。不回避困难，不积累矛盾，勇于担当，敢于负责，务实高效地推进各项工作。坚持勤勉敬业，执政为民，始终保持奋发有为的精神状态。加强绩效管理，严格行政问责，确保政令畅通。深化行政审批制度改革，减少办事环节，改进涉企服务，积极推进行政便利化。

　　要努力建设风清气正的廉洁政府。严格落实党风廉政建设责任制，强化教育，注重预防，健全机制，约束行政权力，规范行政行为。切实强化资金使用、工程招标、土地出让、政府采购和资产处置等方面监管，支持监察、审计部门依法独立履行职责。厉行勤俭节约，反对奢侈浪费，建设节约型机关。加强机关作风建设，以优良政风促行风带民风，努力建设人民满意政府。

　　各位代表，辽源正在崛起，前景催人奋进。让我们在市委的正确领导下，解放思想，开拓创新，团结拼搏，扎实工作，为全市人民生活得更加美好而努力奋斗！
